# Disturbance of the Glutamate-Glutamine Cycle, Secondary to Hepatic Damage, Compromises Memory Function

**DOI:** 10.3389/fnins.2021.578922

**Published:** 2021-01-27

**Authors:** Ilhuicamina Daniel Limón, Isael Angulo-Cruz, Lesli Sánchez-Abdon, Aleidy Patricio-Martínez

**Affiliations:** ^1^Laboratorio de Neurofarmacología, Benemérita Universidad Autónoma de Puebla, Puebla, Mexico; ^2^Facultad de Ciencias Biológicas, Benemérita Universidad Autónoma de Puebla, Puebla, Mexico

**Keywords:** hyperammonemia, hepatic encephalopathy, learning and memory, liver, neuroinflammation

## Abstract

Glutamate fulfils many vital functions both at a peripheral level and in the central nervous system (CNS). However, hyperammonemia and hepatic failure induce alterations in glutamatergic neurotransmission, which may be the main cause of hepatic encephalopathy (HE), an imbalance which may explain damage to both learning and memory. Cognitive and motor alterations in hyperammonemia may be caused by a deregulation of the glutamate-glutamine cycle, particularly in astrocytes, due to the blocking of the glutamate excitatory amino-acid transporters 1 and 2 (EAAT1, EAAT2). Excess extracellular glutamate triggers mechanisms involving astrocyte-mediated inflammation, including the release of Ca^2+^-dependent glutamate from astrocytes, the appearance of excitotoxicity, the formation of reactive oxygen species (ROS), and cell damage. Glutamate re-uptake not only prevents excitotoxicity, but also acts as a vital component in synaptic plasticity and function. The present review outlines the evidence of the relationship between hepatic damage, such as that occurring in HE and hyperammonemia, and changes in glutamine synthetase function, which increase glutamate concentrations in the CNS. These conditions produce dysfunction in neuronal communication. The present review also includes data indicating that hyperammonemia is related to the release of a high level of pro-inflammatory factors, such as interleukin-6, by astrocytes. This neuroinflammatory condition alters the function of the membrane receptors, such as N-methyl-D-aspartate (NMDA), (α-amino-3-hydroxy-5-methyl-4-isoxazolepropionic acid) AMPA, and γ-aminobutyric acid (GABA), thus affecting learning and spatial memory. Data indicates that learning and spatial memory, as well as discriminatory or other information acquisition processes in the CNS, are damaged by the appearance of hyperammonemia and, moreover, are associated with a reduction in the production of cyclic guanosine monophosphate (cGMP). Therefore, increased levels of pharmacologically controlled cGMP may be used as a therapeutic tool for improving learning and memory in patients with HE, hyperammonemia, cerebral oedema, or reduced intellectual capacity.

## The Glutamate-Glutamine Cycle Is a Process Involved in Regulating Excessive Ammonia Concentrations

Ammonia is an important source of nitrogen for living systems, as it is required for the synthesis of amino acids, which form the basis of proteins. Ammonia is both a final and intermediate metabolic product of various biochemical pathways in the body and must be removed as quickly as possible, given its high toxicity. Normal serum ammonia concentrations range from 20 to 50 μM, with a 400 μM increase in circulating ammonia concentrations causing alkalosis and neurotoxicity. A weak base, ammonia (NH_3_) is in equilibrium with the ammonium ion (NH_4_^+^, weak acid), while, in body fluids with a physiological pH (∼7.4), more than 98% of the ammonia is present as NH_4_^+^ (Bromberg et al., 1960; Felipo and Butterworth, 2002). As a weak base, ammonia is easily transported, via diffusion, through the phospholipid bilayer of the cell membranes, including the blood-brain barrier. However, as a weak acid, ammonia is also able to enter the brain to levels of up to 25%, with both of its forms, NH_3_ and NH_4_^+^, exerting toxic effects by altering intracellular and extracellular pH (Erickson, 1985; Felipo and Butterworth, 2002; Bosoi and Rose, 2009). Interestingly, it has been shown that NH_4_^+^ has ionic properties very similar to those of K^+^, which enable it to compete with ionic membrane channels, such as the K^+^ channels, the ATPase transporters Na^+^/K^+^, and the Na^+^/K^+^/2Cl^–^ cotransporter (Moser, 1987; Jayakumar and Norenberg, 2010; Kelly et al., 2009).

**FIGURE 1:**
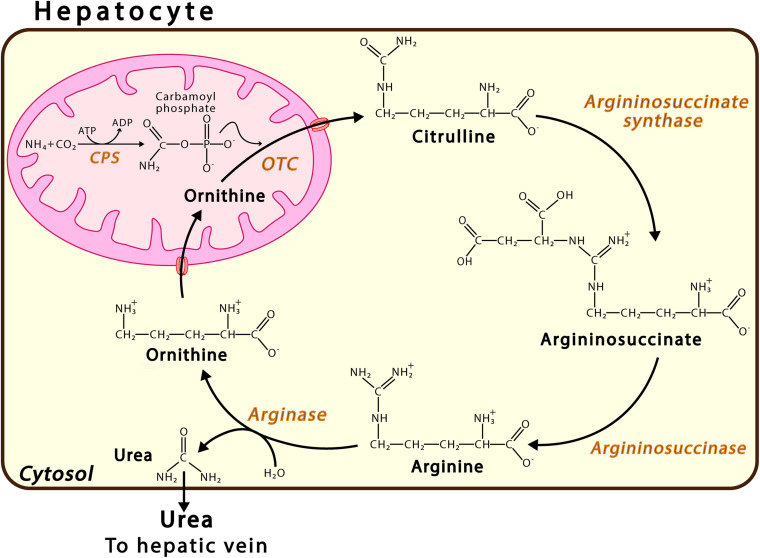
Urea Cycle. This pathway takes place in the periportal hepatocytes. Urea synthesis proceeds via a sequence of six enzymatic reactions, the first three located in the mitochondrion and the last three in the cytosol. The continuity of this process also requires transport proteins in the mitochondrial membrane, such as ORNT1, which enables ornithine (Orn) import, and citrulline, carbamoyl phosphate synthase; and OTC, ornithine transcarbamylase.

Ammonia is produced in mammals by at least 20 metabolic reactions involving both enzymes, such as glutaminase and glutamate dehydrogenase, and purine nucleotide cycle pathways (Yudkoff et al., 1986; Hertz and Schousboe, 1988; Zielke et al., 1990; Frandsen and Schousboe, 1993; Dimski, 1994). In adult humans, a high-normal protein intake produces approximately 1,000 mmol ammonia daily (Atkinson and Camein, 1982; Atkinson and Bourke, 1984; Walker, 2014). The main route of ammonia detoxification occurs via the synthesis of urea in the liver (Figure 1), wherein urea is produced in the liver, travels through the hepatic vein to the kidneys, and is excreted via urine. The failure of liver function that occurs due to hepatic cirrhosis or other conditions may result in an uncontrolled increase in the levels of ammonia in the circulating blood (Clemmensen et al., 1999; Oja et al., 2016). While, in the liver, NH_4_^+^ is converted into urea, this cannot occur in the brain, where, instead, ammonia is incorporated, and thus detoxified, into the amide group of glutamine. For years, the main ammonia detoxification pathway has been thought to operate via the synthesis of urea in the liver, occurring in the periportal hepatocytes (Figure 1). However, evidence shows that hepatic glutamine synthetase contributes to both enteral and systemic ammonia detoxification (Häussinger, 1983; Häussinger and Schliess, 2007; Zhou et al., 2020). Excess ammonia that is not used in the synthesis of urea is absorbed with high affinity by a small population of perivenous hepatocytes, which detoxify ammonia via the amidation of glutamate, thus forming glutamine (Häussinger and Schliess, 2007). The nervous system synthetizes glutamine in order to eliminate the neurotransmitter glutamate, indicating the key role played by glutamine synthetase in neurotransmission (Suarez et al., 2002). Research conducted on glutamine synthetase in the liver of knock-out mice found that the phased increase of enteral ammonia infusion revealed 35% ammonia detoxification via hepatic glutamine synthetase and 35% via urea cycle enzymes, while 30% of ammonia is degraded in other parts of the body (Hakvoort et al., 2017). It has been proposed that the glutamine synthetase reaction in the perivenous hepatocytes acts as a high-affinity low-capacity ammonia clearance system, independent of the urea cycle pathway (Zhou et al., 2020), which itself acts as a low-affinity high-capacity ammonia clearance system located in the periportal hepatocytes. Dysfunction in either the urea or glutamine synthetase cycle triggers disorders such as hyperammonemia and hepatic encephalopathy (Qvartskhava et al., 2015; Hakvoort et al., 2017).

**FIGURE 2:**
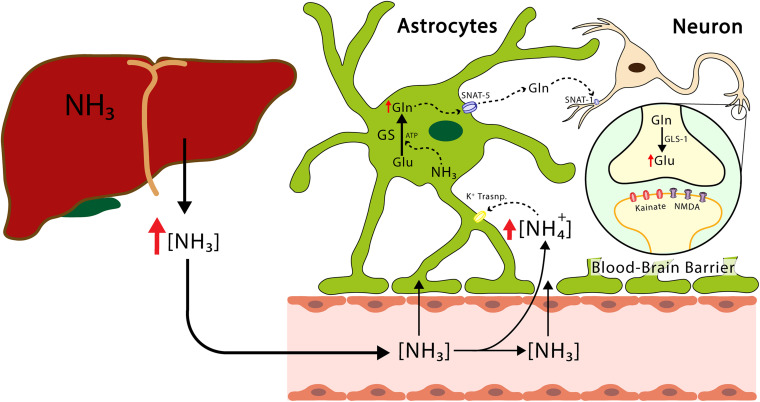
The transport of ammonium from the liver to the CNS. The ammonium leaves the liver and reaches the CNS, crosses the blood-brain barrier, reaches the astrocytes, and produces the release of proinflammatory factors and glutamine. The latter enters the neuron and increases the concentration of glutamate. Excess hyperammonemia and glutamate will be triggers of neuronal damage. GS, glutamine synthetase; GLS-1, phosphate-activated glutaminase; Glu, glutamate; Gln, glutamine; NH_3_, ammonia; NH_4_^+^, ammonium ion; NMDA, N-Metil-D Aspartate receptor; SNAT-1, astrocytic glutamine transporter-1.

Glutamine synthetase is located mainly, but not exclusively, in the astrocytes. Interestingly, it has been shown that the enzyme glutamine synthetase is also found in oligodendrocytes, glial cells known to produce myelin and ensheathing axons in the central nervous system, in caudal regions such as the midbrain and the spinal cord (Pan and Chan, 2017). It has been proposed that while the deletion of glutamine synthetase specific to rat oligodendrocytes does not alter myelination, it does alter neuronal glutamatergic transmission (Xin et al., 2019). While these findings are controversial, they are of physiological interest. Recently synthetized glutamine is released from the astrocytes and transferred to the neurons, where glutamate is regenerated by means of phosphate-activated glutaminase (GLS-1) an enzyme found in abundance in the neurons and participating in the glutamate-glutamine cycle (Botman et al., 2014; Figure 2). This cycle plays an important role in glutamate homeostasis, a process occurring in the brain, by preventing an excess of this amino acid and, thus, excitotoxicity. The accumulation of excess extracellular glutamate has been observed when the brain is exposed to ammonia, although high concentrations of ammonia have also been shown to depolarize hippocampal neurons and induce morphological modifications in astrocytes and neurons (Gregorios et al., 1985; Fan and Szerb, 1993; Bosoi and Rose, 2009). As neurons lack the enzymes necessary for the *de novo* synthesis of glutamate and GABA neurotransmitters from glucose, glutamine serves as a source of carbon for the repletion of glutamate and GABA (Shank and Campbell, 1984). Studies conducted on both rodents and humans have shown that the cyclical flow of glutamine/glutamate is necessary for replenishing both glutamate and GABA and is responsible for 80% of net glutamine synthesis (Kanamori et al., 2002).

The glutamate-glutamine cycle plays an important role in the homeostasis of glutamate levels, a process occurring in the brain to prevent an excess of this amino acid and, thus, excitotoxicity. Thus, the storage of glutamine in the astrocyte would act as a pool for the synthesis of not only glutamate but also GABA (Schousboe et al., 2014). As discussed above, astrocytes are clearly capable of synthetizing and using glutamine, and, although they undoubtedly supply glutamine to the neurons, these cells also contribute to meeting the physiological need of metabolizing this compound (Yudkoff et al., 1988). Therefore, astrocytes play an important role in both the synthesis and metabolism of glutamine, in order to maintain corresponding physiological levels (Yudkoff et al., 1988). It has also been shown that the glutamate-glutamine cycle does not correspond to a stoichiometric reaction, given that not all glutamate released by the neurons is recaptured by the astrocytes for glutamine synthesis (McKenna, 2007, 2012). During this process, some glutamate is lost via intermediaries in other pathways involved in the energy metabolism, such as the TCA cycle, or in the synthesis of the neurotransmitter GABA. These processes play an important role in not only the homeostasis of the synthesis of both glutamate and GABA, but also of cell metabolism and, thus, function.

### Hyperammonemia

**FIGURE 3:**
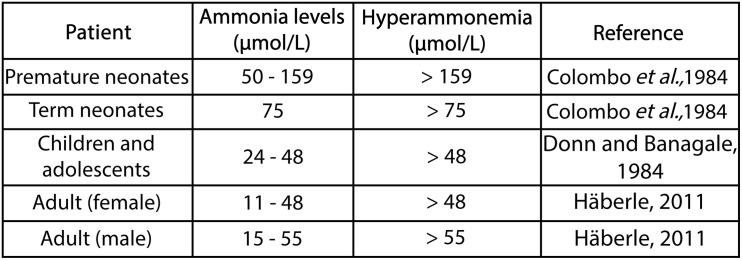
Ammonia concentration serum in different patients.

Ammonia is normally found in small amounts in the blood, while its concentration depends on the age of the patient (Figure 3; Donn and Banagale, 1984). Hyperammonemia is a clinical condition that occurs due to blood ammonia increases greater than 50 μmol/L in adults and greater than 100 μmol/L in newborns (Colombo et al., 1984; Häberle, 2013). The most common clinical cause of hyperammonemia is impaired liver function resulting from such conditions as liver cirrhosis and acute and chronic liver failure. However, it also can occur due to the excessive production of ammonia (caused by fulminant liver failure, intestinal bacterial growth, and some drugs) or due to problems in the detoxification of ammonia (caused by urea cycle disorders in which there is a defect in any of the enzymes involved or decreased glutamine synthetase activity) (Sunheimer et al., 1994; Leonard and Morris, 2002; Vasconcelos et al., 2007; Sundaram and Shaikh, 2009; Lewis et al., 2012). Consequently, hyperammonemia can be classified as either primary or secondary, where the former occurs if the urea cycle is directly affected by any of the enzymes or transporters involved in the cycle. However, if urea cycle function is inhibited due to either an accumulation of metabolites or substrate deficiencies, the resulting increase in ammonia induces secondary hyperammonemia. This distinction is useful for understanding the cellular, biochemical, and molecular mechanisms involved in hyperammonemia and informs the search for and choice of new diagnostic and therapeutic methods (Leonard and Morris, 2002; Häberle, 2011; Natesan et al., 2016). However, both types of hyperammonemia can cause hyperammonemic encephalopathy and irreversible brain damage if they are not treated in a timely manner. In general terms, hyperammonemia can cause muscle, kidney, lung, and liver damage, although it should be noted that the most susceptible organ is the brain – more during development than once the subject has reached adulthood. Hyperammonemia is a factor contributing to abnormalities in the blood brain barrier (Ziylan et al., 1993; Felipo, 2008). The effects of this condition on the brain depend on several factors, such as the stage of brain development, with evidence showing that the toxic effects on the brain are particularly pronounced during human infancy and early childhood development, data which have also been found in pre and postnatal rats (Msall et al., 1984; Aguilar et al., 2000). Other factors determining the effects of hyperammonemia may be the concentration of ammonia, the speed at which ammonia levels increase, the clinical course (either acute or chronic hyperammonemia), and the presence of other clinical alterations, such as peripheral inflammation or neuroinflammation. Depending on the factors mentioned above, hyperammonemia can cause irreversible damage to the brain, of varying degrees of severity, potentially causing intellectual disability in infants, children, and adolescents.

Hyperammonemia with ammonium levels higher than 400–500 μmol/L increases the risk of irreversible brain damage from five to tenfold. The signs and symptoms of hyperammonemia are usually neurological in nature and range from mild cognitive and psychomotor alterations to impaired intellectual functioning, personality changes, altered levels of consciousness, and neuromuscular dysfunction (Dhiman and Chawla, 2008). Hyperammonemia can also trigger severe cerebral edema, brain stem herniation, HE, cognitive impairment, seizures, and cerebral palsy, and, in much more severe cases, may cause neurodevelopmental intellectual disability and even death (Cagnon and Braissant, 2007; Häberle, 2013). Hyperammonemia seems to affect brain function via several mechanisms, with astrocytes the cells primarily affected, due to their topographical position in the blood-brain barrier (Albrecht, 2007), with astrocyte damage, known as astrocytic swelling, playing a key role in brain damage. Astrocytes play an important role in the maintenance of CNS function by means of their interaction with other neural cells, such as neurons and endothelial cells, and their modulation of both excitatory and inhibitory neurotransmission. One of the mechanisms known to be produced by hyperammonemia is a bioenergetic failure that can be caused by increased ATP consumption in astrocytes due to increased glutamine synthesis, enhanced Na-K-ATPase activity, and even the inhibition of enzymes within the tricarboxylic acid cycle (TCA cycle) (Farinelli and Nicklas, 1992; Waniewski, 1992; Albrecht, 2007; Albrecht et al., 2009).

While increased glutamine synthesis occurs in response to increased ammonia levels in the brain, hyperammonemia exceeds the brain’s capacity to synthesize glutamine, causing ammonia concentrations to rise significantly. One way in which ammonia can be eliminated in the brain is via glutamine synthesis resulting from the activation of astrocytic glutamine synthetase; therefore, the neuron does not have an effective defense mechanism against increased ammonia levels transmitted by the blood and depends on the neighboring astrocyte for the elimination of ammonia. Glutamine synthetase is not induced in the brain during chronic hyperammonemia, thus exacerbating the vulnerability of the neuron to increased ammonia levels, with reduced glutamine synthetase activity presenting in the cerebellum and cerebral cortex in rats with portacaval anastomosis (Desjardins et al., 1999). Hyperammonemia has been shown to trigger inflammation and protein nitration in astrocytes (Häussinger et al., 2005), a finding observed after the administration of ammonium in animal models. The colocalization between the immunoreactivity of nitrotyrosine and the GFAP protein of the glia is found along the length of the of blood vessel, providing evidence that protein nitration damages the blood-brain barrier (Schliess et al., 2002; Gorg et al., 2003), an effect also found in rats with portocaval anastomosis (Häussinger et al., 2005).

Coupled with this, an increased of glutamine can mediate key aspects of ammonia neurotoxicity. Glutamine has an osmotic effect on astrocytes and, moreover, having been shown to accumulate in astrocytic mitochondria in the presence of elevated ammonia levels (Dolińska et al., 1996), can cause the mitochondrial dysfunction that mediates the generation of free radicals and contributes to oxidative damage. Ammonia inhibits the neuron-astrocyte trafficking conducted by glutamate and affects postsynaptic glutamate receptors (Butterworth, 1993), while hyperammonemia also affects other neurotransmitter systems in the brain, such as the synthesis of histamine, serotonin, dopamine, and noradrenaline (Hawkins and Mans, 1994). Other mechanisms implicated in ammonia toxicity are systemic inflammation and neuroinflammation. Both human and experimental studies in rodents have been shown to activate astrocytes and M1 microglia, and, even, increase the production of proinflammatory cytokines, such as interleukin 1 beta (IL-1β), interleukin 6 (IL-6), interleukin 8 (IL-8), and α TNF, in both serum and the brain (Montoliu et al., 2009; Rodrigo et al., 2010). This leads to increased blood-brain barrier permeability and contributes to brain damage.

### Hepatic Encephalopathy

Hepatic encephalopathy with subclinical and clinical symptoms is a serious complication of acute or chronic liver damage. The occurrence of a number of abnormalities in the brain, followed by liver damage, are psychoneurological symptoms of HE and include drowsiness and excitement, confusion, and coma, while cerebral edema often presents with intracranial hypertension and morphological changes that lead to functional changes (Blendis, 2006). Portocaval anastomosis with the presence of ammonia has been associated with the fitting of a bowel shunt, leading to an increase in ammonia concentration typical of HE, while high blood flow rate and high ammonium levels are implicated in the pathogenesis of this condition. In addition to being an etiological factor in HE, hyperammonemia is related to other pathologies, such as dysfunctions in the urea cycle of newborns, Reye’s syndrome, toxicity from the ingestion of valproic acid, and idiopathic hyperammonemia (Record, 1991; Brusilow, 2002). Therefore, high concentrations of glutamine and ammonia, its main metabolite, cause cerebral damage, including the failure of different neurotransmission systems, bioenergetic alterations, and oxidative stress. Since the 1950s, research has been conducted on the possibility of establishing a relationship between ammonia concentrations in the blood and neurological disorders; however, there are discrepancies between these events. While cirrhotic patients with high levels of ammonia in their blood were found to present neurological disorders, the concentration of ammonia in the blood was not consistent in patients with chronic hepatic encephalopathy (Phear et al., 1955; Shawcross et al., 2011).

A study conducted by Ong et al. (2003) found that 60% of patients without symptoms of hepatic encephalopathy presented high levels of ammonia, while patients with grade 3 or 4 hepatic encephalopathy presented normal or slightly elevated levels of ammonia in the blood. This finding suggests that, further to the toxicity of ammonia, there are other determining factors in triggering hepatic encephalopathy, such as inflammation, which has been proposed as an important factor (Aldridge et al., 2015). Clinical studies have examined the importance of the inflammation and infection to which cirrhotic patients are prone, with both processes participating in the modulation of the symptoms of chronic hepatic encephalopathy, thus indicating, in the majority of cases, a poor prognosis (Shawcross et al., 2011).

Muscular disorders, such as tremors or asterixis, and electroencephalographic changes with the presence of theta waves, occur in HE. When HE presents with cirrhosis, psychomotor damage causing increased response times, sensory changes, low concentration, memory damage, personality changes (although the mechanism responsible is not yet clear) (Weissenborn et al., 2003), and the presence of memory disorders are all observed.

The main risk factors for HE are acute or chronic liver damage, while another important factor is that ammonia reaches the systemic circulation directly, due to a hepatic portal shut down in which the circulation of the digestive tract bypasses the liver, thus reaching the general circulation directly. The mechanisms that cause this cerebral dysfunction remain largely nuclear, with HE classified into three types, according to the underlying hepatic condition: Type A, which is associated with acute liver failure; Type B, which presents in patients with portosystemic bypass and without intrinsic hepatocellular disease; and, Type C, which is associated with cirrhosis, portal hypertension, or portosystemic stent-shunts and is sub-divided into three categories – episodic HE (precipitated, spontaneous, and recurrent), persistent HE (light, severe, and treatment dependent), and minimal HE (Ferenci et al., 2002). In terms of the presence of HE causing mental damage and impairing the patient’s state of consciousness, low-intensity neurological and psychiatric damage is observed, along with damage to working memory and attention, psychomotor agitation, visuospatial changes, and electroencephalographic changes.

Moreover, HE is classified into the following stages: confusion; lethargy; stupor; and, coma (Lockwood, 2000; Montagnese et al., 2013). The confusion stage is characterized by a state of light damage, along with mood swings, attention deficit, and difficulty forming ideas. The lethargy stage is characterized by temporary disorientation, making it difficult to organize ideas and causing personality changes. In the stupor stage, the patient finds it impossible to perform mental tasks, experiencing space and time disorientation, amnesia, and psychomotor agitation. Finally, in the coma stage, decerebration occurs, a state which is practically irreversible (Vilstrup et al., 2014).

## Hepatic Encephalopathy and Hyperammonemia Produce Neuronal Communication Dysfunction in the CNS

Glutamate is the main excitatory neurotransmitter in the CNS and is the precursor for GABA, the main inhibitory neurotransmitter of the CNS (Hayashi, 1952; Curtis and Watkins, 1960). Glutamate is synthetized in the synaptic terminal of the glutamatergic neurons and stored in synaptic vesicles to be subsequently released into the synaptic cleft via a mechanism involving the N- and P/Q-type voltage-gated Ca^2+^ channels (Birnbaumer et al., 1994). Once released, glutamate activates ionotropic and metabotropic glutamatergic receptors (iGluR y mGluR, respectively), with the former comprising NMDA, AMPA, and kainate receptors. The twelve types of mGluR are classified into three groups, according to their pharmacology: Group I, which comprises mGluRs 1 and 5; Grupo II, which comprises mGluRs 2 and 3; and, Grupo III, which comprises the remaining mGluR types (Reiner and Levitz, 2018). Despite the importance of glutamate as a neurotransmitter in the CNS, its excessive release may be toxic for the brain and may cause excitotoxicity. However, the excitotoxicity of glutamate has been associated with exposure to severe stress and neurodegenerative disease (Gilad et al., 1990; Lewerenz and Maher, 2015). The excessive release and absorption of glutamate has been identified in brain regions, such as the frontal cortex and the hippocampus, of rats exposed to various forms of stress (Moghaddam, 1993). Excess extracellular glutamate triggers mechanisms that involve astrocyte-mediated inflammation, including the release of Ca^2+^-dependent glutamate by astrocytes and the inhibition of glutamate re-uptake via the inhibition of glutamate transporter (Mahmoud et al., 2019). Glutamate re-uptake not only prevents excitotoxicity, but also acts as a vital component in synaptic plasticity and function (Nadler, 2012).

To date, five sub-types of glutamate transporters, known as excitatory amino acid transporters (EAATs 1–5), have been identified in the plasma membrane of different mammals (Danbolt, 2001). There are two types of sodium-dependent transporters in the astrocyte: EAAT1 (GLutamate ASpartate Transporter -GLAST- in rodents), which is mainly an astroglial transporter and is the main transport protein present during the development of the CNS (Furuta et al., 1997); and, EAAT2 (GLT-1 in rodents) (Lehre et al., 1995), which is an astroglial transporter expressed postnatally in all glutamate transport occurring in mature tissue (Tanaka et al., 1997; Danbolt, 2001). Moreover, EAAT3 (known as EAAC1 in rodents) is found in the postsynaptic neural membranes of the hippocampus, the cerebellum, and the basal ganglia, although at a much lower density than EAAT1, which is found in the astrocytes (Holmseth et al., 2012). EAAT4 is mainly found in the Purkinje cells of the cerebellum (Bar-Peled et al., 1997; Nagao et al., 1997; Dehnes et al., 1998), while EAAT5 is mainly found in the photoreceptors of the retina and bipolar cells (Arriza et al., 1997; Pow and Barnett, 2000).

**FIGURE 4:**
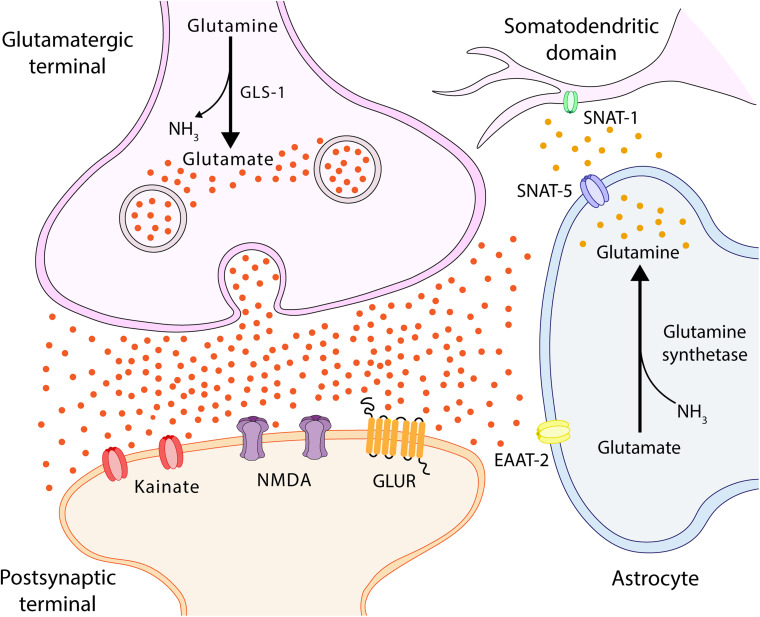
Glutamate-glutamine cycle. Ammonia from the blood is neutralized in the astrocyte via a reaction with glutamate (Glu), which uses glutamine synthetase to form glutamine (Gln). Gln is transported by SNAT-5 to the extracellular environment, where it can then be transported to the cerebrospinal fluid (CSF) or transferred to neurons by SNAT-1, located in the somatodendritic domain (Melone, 2004; Conti and Melone, 2006). In the neuron, Gln is degraded by phosphate-activated glutaminase (GLS-1) into Glu and ammonia. Glu feeds the neurotransmitter pool and is released into the synaptic cleft, interacting with Glu receptors located in both synapses and astrocytes. SNAT-1, neuronal glutamine transporter; SNAT-5, astrocytic glutamine transporter-5; and EAAT, excitatory amino acid transporter.

The membrane potential of the astrocytes is much lower than that of the neuronal membranes, thus facilitating glutamate re-uptake by the astrocyte (Allert et al., 1998; Figure 4). The high concentrations of ammonia are able to increase membrane potential, thus depolarizing both neurons and astrocytes. Astrocytes, which have a lower resting membrane potential than neurons, present an increase in said levels depending on the concentration of membrane potential subsequent to the application of 10 and 20 mM NH4Cl 5 (Allert et al., 1998). It has been shown that the activation of the ionotropic glutamate receptor NMDA plays a significant role in pathophysiology of HE (Llansola et al., 2007). The opening of the channel is controlled by a powerful voltage-dependent channel block comprising external magnesium ions (Mayer et al., 1984; Nowak et al., 1984). It is believed that, by increasing the membrane potential, ammonia eliminates the magnesium block, leaving the NMDA receptors susceptible to activation. The point at which ammonia eliminates the magnesium block remains to be established, as it has been shown that half of it is eliminated when the membrane potential rises to −20 mV (Mayer et al., 1984). In conclusion, while the physiopathological concentrations of ammonia directly increase the membrane potential of both astrocytes and neurons, this is not sufficient to activate voltage-dependent channels or generate action potential in neurons. It should be noted that glutamine synthesis varies in different pathological conditions, causing changes to the mitochondrial permeability of astrocytes via glutamine deamination, which, in turn, causes an increase in intramitochondrial ammonia and, consequently, results in a toxic environment for mitochondria (Natesan et al., 2016).

Acute liver failure has been found to damage the genes expressing glial fibrillary acidic protein (GFAP), which participates in the mobility and structure and provides structural stability (Belanger et al., 2002). The intensity of the damage to the genes is related to the severity of hyperammonemia and cerebral oedema in animals with ischemia/ischemic animals, while an oedema was found when ammonium was added to primary astrocyte cultures (Norenberg et al., 1990). An impressive range of amino acid transporters, such as the glutamate transporter EAAT 2 and the glycine transporter GlyT-1, have been found in the astrocyte membrane. Ammonium alters glutamate re-uptake in the astrocyte, with *in vitro* and *in vivo* studies observing that this is caused by the down-regulation of the transporters EAAT-1 and EAAT-2 (Knecht et al., 1997; Norenberg et al., 1997). The expression of ARNm and the transporter protein for EAAT-1 and EAAT-2 decrease in acute hyperammonemia (Knecht et al., 1997; Chan and Butterworth, 1999; Felipo and Butterworth, 2002), while extracellular glutamate and glycine concentration increase, generating a neurotoxic environment (Michalak et al., 1998). Levels of EAAT-2 transporter have been found to decrease in different areas of the brain in acute liver failure (Knecht et al., 1997), which leads to elevated glutamate concentrations in the synaptic space, thus suggesting that reduced levels of this transporter contribute to the different degrees of oedema observed in encephalopathy. A decrease in the levels of the transporters EAAT-1 and EAAT-2 has not been consistently shown in chronic hyperammonemia, while a significant decrease has been observed in the levels of the NMDA receptor, as described above.

Ammonium inhibits the oxidation of glucose into pyruvate (Lai and Cooper, 1986), stimulates glycolysis, and alters mitochondrial function (Bai et al., 2001). Moreover, it has been shown that chronic ammonium exposure in cultures of the granule neurons of the cerebellum induces the redistribution of protein kinase C (PKC) isoforms in the membrane and cytosol, thus causing cell dysfunction (Giordano et al., 2005). These changes in glutamatergic neurotransmission are related to long-term ammonia exposure, which is associated with alterations in the phosphorylation of NMDA receptors mediated by PKC (Monfort et al., 2002). It should be noted that mortality from acute ammonia toxicity is avoided via the administration of NMDA receptor antagonists (Marcaida et al., 1992; Hermenegildo et al., 1996). Acute ammonia exposure results in the activation of both NMDA receptor and the cyclic GMP pathway, leading to increased nitric oxide levels in the brain and, thus, promoting an oxidative environment and neuronal degeneration.

Interestingly, it has been demonstrated that different types of glutamate receptors are affected in both acute and chronic hyperammonemia. Glutamine synthesis increases *in vivo* during acute hyperammonemia, acting as a mechanism for detoxifying excess ammonia, and induces the excessive activation of NMDA receptor, which causes death via acute ammonia poisoning (Marcaida et al., 1992; Hermenegildo et al., 2000). Chronic hyperammonemia affects the signal transduction pathways for the NMDA I group, AMPA, and the mGluR receptors, contributing to the development of various cognitive or motor disorders involving specific areas and circuits (Canales et al., 2003; Llansola et al., 2007). Experimental models have revealed that the prolonged survival of the cortical neuron occurs in hyperammonemic animals administered with antagonists of the NMDA receptor, such as MK-801 and 2-amino-5-phosphonovaleric acid (APV) (Kosenko et al., 1999; Rama Rao and Qureshi, 1999).

**FIGURE 5:**
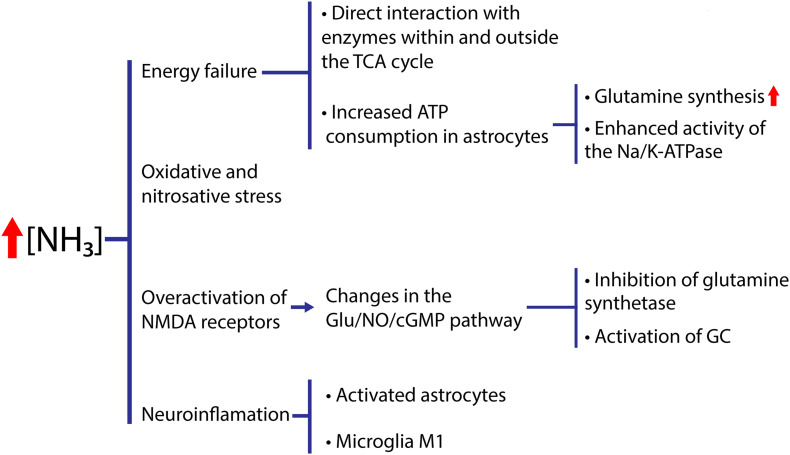
Hyperammonemia causes neurotoxicity. Energetic dysfunction, increased nitrosidative stress and neuroinflammation occur. These factors cause a neurotoxic environment for the neuron and will be a consequence of cognitive damage.

In acute hyperammonemia, the activation of NMDA receptors increases the ingress of sodium and Ca^2+^, with the latter activating calcineurin, which dephosphorylates and activates Na + /K + ATPase, which, in turn, extracts sodium and increases the ingestion of ATP, while the mitochondria absorb the excess calcium (Carvalho et al., 2016). Mitochondrial dysfunction and the generation of ROS are both involved in the neuronal alteration occurring in acute hyperammonemia (Natesan et al., 2016), reducing ATP synthesis in mitochondria by altering the homeostasis of Ca^2+^. This mitochondrial dysfunction and the neurotoxic environment in cytosol generate dysfunction in different parts of the brain. Pre-treatment with NMDA receptor antagonists completely prevents ammonia-induced increases in Na + /K + -ATPase activity (Ott et al., 2005). The deficit caused by acute ammonia poisoning affects various cerebral processes (Braissant et al., 2012; Figure 5).

## Hyperammonemia Causes Neuroinflammation and Alters Receptors Such as N-Metil-D-Aspartate (NMDA) and AMPA, Which Are Responsible for Altering Learning and Spatial Memory

Chronic or acute liver/hepatic damage may cause different degrees of HE due to the action of various toxic substances, such as ammonia, which may enter the brain and alter its function. Hepatic encephalopathy is a clinical condition caused mainly by liver/hepatic damage, although it can also occur without the presence of said damage (Jayakumar and Norenberg, 2018). Various studies have shown that HE is associated with cognitive deterioration, with, particularly, hyperammonemia negatively affecting the learning process and working memory (Elithorn et al., 1975; Aguilar et al., 2000). Both hyperammonemia and hepatic/liver failure induce alterations in glutamatergic neurotransmission, which may be the main cause of HE (Felipo and Butterworth, 2002; Monfort et al., 2002). This imbalance may explain the damage to both learning and memory, due to the fact that the glutamatergic receptors are present in both neurons and astrocytes, cells which play a very important role in learning and memory processes.

**FIGURE 6:**
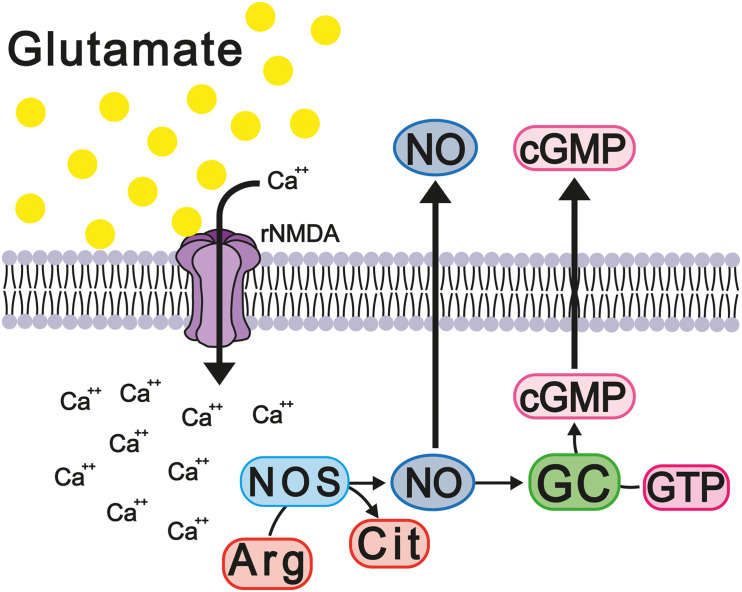
Hyperammonemia causes deregulation of the glutamate-NO-cGMP cycle. These changes increase the release of proinflammatory factors and neuroinflammation with memory damage.

It is well reported that the constant activation of the NMDA and AMPA receptors modifies the homeostasis of intracellular calcium, thus modifying its function as a second messenger and major modulator of calcium-dependent enzymes, such as calmodulin and nitric oxide synthases 1 and 2 (NOS-1 and NOS-2). Calcium binds with calmodulin and activates NOS, increasing the levels of nitric oxide (NO), which, in turn, activates soluble guanylate cyclase, thus increasing cGMP concentration. The latter messenger activates the cGMP-dependent protein kinase (PKG) that phosphorylates and activates cGMP-specific phosphodiesterase 5 (PDE-5), which degrades excess cGMP (Figure 6). It should be noted that the activation of the glutamate-nitric oxide-cGMP pathway modulates different cerebral processes, such as the sleep-wake cycle, long-term potentiation (LTP), and some types of learning and memory (Ding et al., 1994; Boulton et al., 1995; Sistiaga et al., 1997).

A process ensuring the lasting improvement of the transmission of signals among two (or more) neurons as a result of the synchronous stimulation of both, LTP is considered one of the main cellular mechanisms underlying both learning and memory. Various authors have proposed that, in the hippocampus, LTP plays an important role in different types of learning, such as spatial learning and working memory (Korshunova et al., 2015; Minami et al., 2016). Experimental studies have shown deteriorated LTP in hippocampus sections of intact rats exposed to ammonia (Monfort et al., 2002, 2004, 2005). Another study conducted on rats treated surgically with portacaval shunting, namely the most common chronic HE model, found altered levels of LTP in the hippocampus, alongside the reduced expression of the NMDA and AMPA receptors. Moreover, it has been shown that the activation of the glutamate-NO-cGMP-PKG-PDE pathway is necessary for the re-establishment, induction, and adequate maintenance of LTP in the hippocampus of hyperammonemic rats (Monfort et al., 2007). These findings reinforce the hypothesis that the glutamate-NO-cGMP pathway is indirectly altered by hepatic/liver damage, thus compromising LTP.

While it is known that the cerebellum is a structure involved in motor processes, equally important is its participation in non-motor processes, with attention, perception, language, working memory, and spatial learning among the processes in which the cerebellum is closely involved (Martin et al., 2003). The glutamate-NO-cGMP pathway in the cerebellum has been studied using NMDA receptor blockers, such as dizocilpine, finding a reduction in NOS levels in the cerebellum but not in the hippocampus or the cortex, with said reduction in NOS, and therefore NO, correlating with the deterioration of working memory. These data suggest that the glutamate-NO-cGMP pathway in the cerebellum plays a very important role in this type of memory (Yamada et al., 1996). Interestingly, the activation of soluble guanylate cyclase by NO is also seen at a reduced rate/level in homogenized cerebellums extracted from patients who have died due to HE (Corbalán et al., 2002a,b). These data suggest that alterations in the glutamate-NO-cGMP pathway in the cerebellums of animals with HE also occur in the brains of humans with HE.

Both competitive and non-competitive antagonism of the NMDA receptor have been shown, in various behavioral tasks, to affect learning and the formation of memory in rodents (Parada-Turska and Turski, 1990; Ward et al., 1990; Parada et al., 1992; Maurice et al., 1994). The blocking of NMDA receptors with (2R)-amino-5-phosphonovaleric acid (AP-5), a competitive antagonist, does not affect low frequency synaptic transmission in the hippocampus, but does prevent the induction of LTP in the hippocampus (Collingridge et al., 1983). Dizocilpine, a potent non-competitive antagonist of the NMDA receptor, alters spontaneous alternation behavior in the Y maze test, which is used as a measure of spatial working memory (Maurice et al., 1994). However, it has been found that NO inhibitors such as N(G)-Nitro-L-arginine methyl ester (L-NAME) and 7-Nitroindazole (7-NI) negatively affect the performance of animals during the acquisition of a task, but not during the retention of said task. The measurement of cGMP levels in the rat brain confirmed the participation of NO/cGMP in the deterioration, induced by dizocilpine and L-NAME, of spontaneous alternation behavior. Decreased cGMP levels, but not cAMP levels, were observed after treatments with dizocilpine, L-NAME, and 7-NI, although these treatments presented different effects. Dizocilpine reduces cGMP levels in the cerebellum, but does not affect the cerebral cortex and hippocampus, while L-NAME and 7-NI reduce cGMP levels in the cerebral cortex and hippocampus (Yamada et al., 1996). These effects may be a result of the fact that, in the cerebellum, the NMDA receptors carry out a more tonic regulatory function in the maintenance of cGMP levels than they do in other areas of the brain. Moreover, the homeostatic mechanism in the cerebellum may be less efficient in regulating cGMP levels, given that it has been shown that phosphodiesterase levels in the cerebellum are ten times lower than in other regions of the brain (Greenberg et al., 1978). Therefore, given that dizocilpine treatment decreased cGMP levels in the cerebellum, but not in the cerebral cortex and hippocampus, and that this effect is reversed by cGMP analogs, it can be concluded that the cerebellum, not the cerebral cortex or hippocampus, is the region responsible for spontaneous alternation behavior (Yamada et al., 1996). Therefore, restoring learning and spatial memory requires the normalization of hippocampus function. Subsequent studies evaluated the effects of chronic treatment with a phosphodiesterase 5 inhibitor (sildenafil) in rats surgically treated with portacaval shunting, finding increased IL-1β and TNF-α levels in the hippocampus along with increased levels of the subunits α1 of the GABA-A receptor and GluR2 of the AMPA receptor. The same study also found decreased levels of the GluR1 subunits of the AMPA receptor and the NR1 and NR2a subunits of the NMDA receptor. These data correlate to reduced spatial learning, as evaluated in the Morris water maze. However, treatment with sildenafil normalized both the levels of IL-1β and TNF-α and the expression of the GABA-A, AMPA, and NMDA receptors, as well as re-establishing spatial learning. These results provide evidence that phosphodiesterase 5 inhibitors may be useful for improving cognitive function in HE patients (Hernandez-Rabaza et al., 2015).

Other mechanisms which cause central damage involve constant chronic peripheral inflammation resulting from hyperammonemia and the release of different chemokines, which enable the extravasation of the blood cells of the CNS, thus contributing to neurological disorders such as HE (Rodrigo et al., 2010). These inflammatory processes are broadly related to cognitive and motor damage (Ochoa-Sanchez and Rose, 2018). High levels of TNF-α, IL-1β, and IL-6 have been found in HE patients (Jayakumar et al., 2015), with some studies finding microgliosis in the brains of acute HE patients (Jiang et al., 2009; Dennis et al., 2013) and others reporting changes to both acute and chronic HE (Jayakumar et al., 2015). Interestingly, it has been shown that the brains of HE patients present astrocytosis similar to that observed in Alzheimer’s disease patients (Norenberg, 1981).

**FIGURE 7:**
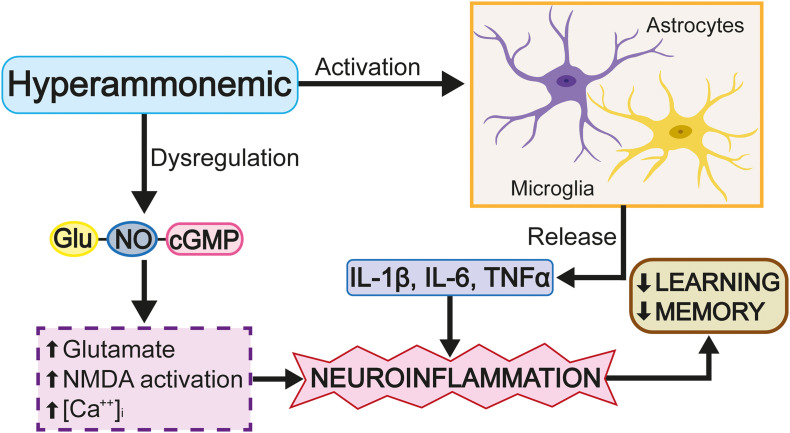
The excess of glutamate activates cGMP. Glutamate activates iNOS, produces nitric oxide (NO) and leads to the activation and extracellular release of cGMP.

Rats surgically treated with portacaval shunting have been shown to present morphological changes in the microglia of the hippocampus, acquiring an ameboid and, consequently, reactive morphology; however, with the application of a phosphodiesterase 5 inhibitor, the level of microglial reactivity reduces (Hernandez-Rabaza et al., 2015). It should be noted that the activation of the microglia is not homogeneous in the entire brain, as some brain regions do not present said activation. Evaluation of the cortex in a portocaval rat model showed that, despite increased ammonium levels in the blood, changes in the cells of the microglia were not observed (Brück et al., 2011). A hyperammonemia model induced by the administration of NH_4_Cl showed that an increased ammonium level in the blood was not able to activate the cells of the microglia in the mouse cortex; however, as the effect of NH_4_Cl is reversible, this only permits the study of the effects of acute increases in ammonium levels (Rangroo Thrane et al., 2012). Chronic hyperammonemia models induced by azoxymethane, which causes irreversible liver damage, have shown that the activation of the microglia in the cortex coincides with the rupture of the blood-brain barrier and the occurrence of cerebral edema (Rangroo Thrane et al., 2012). The discrepancy, discussed above, between the results obtained for the cortex and hippocampus is probably due to the high level of antioxidant enzyme expression in the cortex, which cushions the impact of increased levels of highly oxidizing molecules, thus giving the cortex an advantage over the hippocampus in limiting oxidative stress (Sharma et al., 2013). Other rat HE models show that an increased ammonium concentration induces the release of proinflammatory molecules, such as IL-1β and IL-6, pro-inflammatory cytokines which carry out the following: the modification of GABAa and GluRI expression (Wang et al., 2012); the disruption of the blood-brain barrier (Brett et al., 1995); the alteration of LPT (Murray and Lynch, 1998; Hoshino et al., 2017); the promotion of microgliosis (Chiang et al., 1994) and astrocytosis (Brunello et al., 2000); and, the deterioration of learning and spatial memory (Figure 7; Moore et al., 2009; Wang et al., 2012). Specifically, increased IL-1β levels interrupt GABAergic and glutamatergic neurotransmission in the hippocampus and complicate spatial learning in rats with HE.

The panorama described above enables the identification of important HE markers, among which the alteration of the glutamate-NO-cGMP pathway and neuroinflammation are highlighted, processes which, individually, have been shown to alter glutamatergic and GABAergic neurotransmission. However, to date, an effective alternative treatment for HE has not been identified. By means of the information detailed above, the present study proposes that research resources be invested in the search for effective therapeutic tools for reducing neuroinflammation and finding new strategies for selectively inhibiting phosphodiesterase 5 in order to improve cognitive functions such as learning and memory.

## Diagnosis and Treatment

The process of clinical diagnosis remains unclear, since the diagnosis must be made based on the clinical characteristics of each patient. The magnitude of liver damage that the patient presents and the severity of liver cirrhosis should be reviewed. In addition to testing for the detection of a port-systemic short-circuit. The dysfunctions in HE cause memory impairment, personality changes, and liver or kidney damage, which can more frequently affect senile or immunosuppressed patients (Arias et al., 2014).

An optimal diagnosis of HE is based on clinical tests with either a *n*europsychological and/or neurophysiological basis. The results of both tests are analyzed together for a better diagnosis (Ferenci et al., 2002; Montoliu et al., 2009; Ciećko-Michalska et al., 2012; Kircheis et al., 2014). Neuropsychological tests evaluate the patient’s cognitive function. The most commonly used test is the psychometric hepatic encephalopathy score (PHES), which evaluates visual abilities, processing speed, attention, language, concentration, learning, and memory (Kircheis et al., 2014; Dhiman, 2015a,b), further to taking into account personality changes and hyperosmia (Ciećko-Michalska et al., 2012). One of the tests evaluates psychomotor speed and cognitive variation via an interface between the reaction time for recognizing a colored field and recalling the name of a color previously written down by the subject (Bajaj, 2013; Stinton and Jayakumar, 2013). Neurophysiological tests measure physiological function, often using electroencephalograms to analyze brain function. Cerebral magnetic resonance is also applied to determine the hyperactivity of both the basal ganglia and white matter and to establish whether the changes observed in these brain regions are associated with the oedema occurring in HE and the level of hyperammonemia (Guerit et al., 2009; Su et al., 2015). Cerebral spectroscopy is used to analyze cerebral mass in relation to ammonium, glutamate, or glutamine exposure, as well as the presence of cerebral/brain water. Given that the tests discussed above are not specific diagnostic tools for hepatic/liver dysfunction, the analysis of the results of both neuropsychological and neurophysiological tests is required for an adequate diagnosis.

The treatment of patients with encephalopathy depends on the intensity of the pathology, while the reduction of ammonia is important to reduce the toxic effects of the condition. In addition to the treatments, a series of therapeutic measures should also be established, such as improved nutrition, hydration, aspiration of secretions and protection of the respiratory tract, and treatment of infections that subsequently occur may also be necessary. As mentioned above, the development of HE is related to the concentration of nitrogenous compounds in the digestive tract, which means that the reduction of intestinal ammonia production is vital for its treatment. For this, drugs (non-absorbable disaccharides or antibiotics) have been developed to reduce the production of ammonia by the intestinal flora, while a lower intake of protein in the diet is also recommended. Lactulose (ß-galactoside-fructose) and lactitol (ß-galactoside-sorbitol), non-absorbable synthetic sugars used to treat constipation, are given to reduce the amount of absorbable ammonia (Vilstrup et al., 2014). Both disaccharides work through the same mechanism of action, which increases the incorporation of nitrogenous products present in the intestinal lumen to the bacterial flora and is associated with an acidic pH, due to the bacterial metabolism of short chain fatty acids. This acidity shifts the balance of the ammonia composition toward a higher proportion of ammonium ion, to which the mucosa is impermeable, thus reducing the amount of ammonia exposed to intravascular lumen. The non-absorbable disaccharides facilitate the expulsion of the flora that has incorporated nitrogenous products (Weber and Fresard, 1981).

One of the first antibiotics used to treat encephalopathy and liver dysfunction was the oral administration of neomycin (Fisher and Faloon, 1957), an antibiotic, from the group of non-absorbable aminoglycosides, whose advantage was that it did not generate ototoxicity and kidney. to damage. The use of metronidazole or vancomycin is limited by their toxic effects, and the use of antibiotics in combination with lactulose was also found to be more effective (Tarao et al., 1990). Rifaximin, a no absorbable form of rifamycin, is an antibiotic with effective antibacterial activity in vi One of the first antibiotics used to treat encephalopathy and liver dysfunction was neomycin (Fisher and Faloon, 1957), an orally administered antibiotic from the group of non-absorbable aminoglycosides, which had the advantage of not causing ototoxicity and kidney damage. The use of metronidazole or vancomycin is limited by their toxic effects, and the use of antibiotics in combination with lactulose was also found to be more effective (Tarao et al., 1990). Rifaximin, a non-absorbable form of rifamycin, is an antibiotic with effective antibacterial activity *in vitro* against gram-negative and anaerobic organisms, achieving a significant decrease in bacterial flora after a few days of use (Adachi and DuPont, 2006). Well tolerated by patients, rifaximin has been used successfully for various intestinal pathologies, such as traveler’s diarrhea or acute diverticulitis. As with neomycin, its efficacy in treating acute liver dysfunction is well documented (Mas et al., 2003).

Another therapeutic measure involves combining the increased elimination of ammonia with the administration of anti-inflammatory drugs in order to reduce neuroinflammation (Tofteng and Larsen, 2004; Aldridge et al., 2015). A recently proposed treatment modulates the activation of TLR 4 receptors and then decreases the production of interleukins, such as IL-6 and the tumor necrosis factor TNF alpha. Sildenafil has been proposed as a treatment option, as it reduces neuroinflammation and restores spatial learning in animal models by lowering IL-6 and TNF alpha levels and restoring GABA A and NMDA receptor levels, thus improving glutamatergic and GABAergic communication (Erceg et al., 2005; Arafa and Atteia, 2013). Finally, L-carnitine is used to prevent hyperammonemia-induced cytotoxicity in astrocytes, an approach which functions by reducing ROS levels and improving the regulation of essential amino acids (Wang et al., 2018).

### Final Comment

Liver damage, whether acute or chronic, can cause different degrees of hepatic encephalopathy. Hyperammonemia causes increases in ammonia in the brain and causes a series of neurochemical and structural changes that lead to cellular dysfunction, which has been linked to damage to the memory process. In the present review, data were shown that relate cell damage and astrocyte activation leading to the release of interleukins that causes inflammation. Furthermore, glutamatergic transmission is altered, which leads to an over-activation of NMDA receptors accompanied by blocking glutamate reuptake, and an increase in intracellular calcium concentration. These conditions lead to a consequent cellular and cognitive dysfunction. On the other hand, the disruption of the blood-brain barrier is another factor that leads to hepatic encephalitis and is probably due to the presence of high concentrations of ammonia and the release of interleukins by astrocytes that surround the blood vessels of the blood-brain barrier. Therefore, research focused on the search for new pharmacological alternatives to reduce or prevent hepatic encephalopathy with the presence of hyperammonemia is urgent for the improvement of these patients.

## Author Contributions

IDL, IA-C, LS-A, and AP-M wrote or contributed to the writing of the manuscript. IL was responsible for the conception of the manuscript and approved the final version for publication. IA-C and LS-A made all the figures in this review. All authors contributed to the article and approved the submitted version.

## Conflict of Interest

The authors declare that the research was conducted in the absence of any commercial or financial relationships that could be construed as a potential conflict of interest.
